# Editorial: Postural control, exercise physiology and the balance training—type of exercises, mechanisms and insights

**DOI:** 10.3389/fphys.2023.1149733

**Published:** 2023-02-17

**Authors:** Antonino Patti, Francesco Fischetti, Fatma Nese Sahin, Antonino Bianco

**Affiliations:** ^1^ Sport and Exercise Sciences Research Unit, Department of Psychology, Educational Science and Human Movement, University of Palermo, Palermo, Italy; ^2^ Department of Basic Medical Sciences, Neuroscience and Sense Organs, University of Study of Bari, Bari, Italy; ^3^ Department of Coaching Education, Faculty of Sport Science, Ankara University, Ankara, Türkiye

**Keywords:** physical activity, gait analysis, falls, dynamic postural, fitness

Postural health, today more than ever, is entering a part of everyday life and is associated with the biomechanical analysis of the human body. The ability to obtain or restore a stable state of balance is referred to as postural control (Pollock et al., 2000). Postural control is a complex task, and many factors contribute to good control. The postural control is dependent on peripheral sensory systems and their functioning. In addition, receptors such as vision and the vestibular system have a significant influence on the postural control system. The quality of the performance of this system depends on the integration of visual, vestibular, proprioceptive, and tactile inputs, which have a role in the modulation of muscle tone and strength regulation and ultimately, maintaining balance (Felson et al., 2009; Nieto-Guisado et al., 2022). Being able to maintain a stable upright position throughout one’s life is an important result that significantly affects the quality of life. The specific tests assess different components of balance ability. In 2023, Van Humbeeck, N. et al. Studied postural control in both children and older adults (Van Humbeeck et al., 2023). The authors showed a planar path length and an ellipse area with a U-shaped developmental trajectory. These conclusions confirm the results of Schwesig et al. (2013). The maximum postural stability and better postural control were identified for the age range from 34 to 44 years. The role of training strategies and programs has fundamental importance both in sports to improve performance and in terms of health promotion, such as the prevention of falls in old age (Bianco et al., 2014; Greco et al., 2019a; Greco et al., 2019b; Giustino et al., 2022). The Research Topic currently includes six manuscripts. This Research Topic aimed to analyze the knowledge of the most functional training programs to improve balance and how to use technologies for the evaluation. The muscles’ performances depend largely on the recruitment of the motor units. Aoyama and Kohno analyzed the differences in the recruitment and rate coding of the motor units of the vastus lateralis muscle between postural and voluntary tasks. The authors showed that the firing properties of motor units clearly differ between postural and voluntary muscle contractions. The authors suggested that the differences depended on the fact that the postural task has a lower percentage of high threshold and high-amplitude motor units than the voluntary task (Aoyama and Kohno). Sarto et al. published in this Research Topic a brief cross-sectional study (Sarto et al.). In this study, the authors found a gap in the literature about the presence of few studies investigating the effects of physical activity, or inactivity, on Postural Balance control in populations of different age groups. The author analyzed 86 volunteers to compare the effects of a physically active lifestyle on static and dynamic postural balance control. The authors supported the concept that the physically active improves postural balance control both in the elderly and young with potentially positive effects on the age-related decline of postural balance performance. Moreover, the authors suggest that dynamic analysis seems more sensitive in detecting differences. On the other hand, various diseases induced by poor posture have attracted public attention. Wang et al. aimed to investigate the influence of basic posture transitions on cardiac function and on autonomic nerves (Wang et al.). In this study, the authors showed that posterior extension, lateral flexion, trunk rotation, and trunk forward flexion could influence the heart rate, cardiac function, and autonomic nerves. In addition, they established the average quadratic value of successive differences in heartbeats, high-frequency power (HF), and the ratio of low frequency (LF) to high frequency characterizing vagal activity, decreases in six postures other than the neutral trunk, while LF/HF, which is a parameter of autonomic balance, increases. Balance is one of the skills to be taken care of at an early age. However, the scientific literature suggests conflicting conclusions on age and gender differences in children’s balance ability. Li et al. proposed a study where they investigate the balance ability of preschoolers and determine how moderate it is for age and gender (Li et al.). Six hundred and nineteen preschool children aged 3–6 years have been enrolled in the study, and the static and dynamic balance was assessed. They found that static and dynamic balance improves with age. In addition, older girls displayed better postural stability than boys. The author’s conclusions showed that the balance is influenced by both age and gender. Jia et al. assessed 765 male football players aged 9 to 11. The authors analyzed some components of physical fitness in these young football players (Jia et al.). Sit and reach test, *t*-test, 30 m run test, and vertical jump tests were administered. Age-specific centile estimations were calculated for physical fitness tests. The results of the study showed how the 30 m run-test, and vertical jump test can be predictive of fitness status: overweight football players did significantly worse. In conclusion, the authors suggest to Chinese policymakers a procedure for the creation of a national physical fitness standard (Jia et al.). The latest study featured in this Research Topic but not published in the final version at the time of writing this editorial was conducted by Chai et al. The authors studied the higher risk of falls in subjects with knee osteoarthritis. They showed as these subjects have developed different body weight-bearing strategies to compensate for the disease-related loss of lower extremity strength, range of motion, and balance. However, compensatory strategies could increase the risk of falls and result in more abnormal knee joint loading. In conclusion, this Research Topic provided multidisciplinary Postural health Investigations, focusing on one good quality of life and being able to play sports, both at an amateur and competitive level. The Research Topic highlights the need for further studies in both sports and everyday life contexts. As technology is needed, researchers are encouraged to develop new tests for both static and dynamic postural assessment. Using wearable technologies, with proper validation, to assess postural health should be a focus of future scientific research.
